# Human iPS cell-derived cardiac tissue sheets for functional restoration of infarcted porcine hearts

**DOI:** 10.1371/journal.pone.0201650

**Published:** 2018-08-02

**Authors:** Masanosuke Ishigami, Hidetoshi Masumoto, Takeshi Ikuno, Takayuki Aoki, Masahide Kawatou, Kenji Minakata, Tadashi Ikeda, Ryuzo Sakata, Jun K. Yamashita, Kenji Minatoya

**Affiliations:** 1 Department of Cardiovascular Surgery, Graduate School of Medicine, Kyoto University, Kyoto, Japan; 2 Department of Cardiovascular Surgery, Kobe City Medical Center General Hospital, Kobe, Japan; 3 Department of Cell Growth and Differentiation, Center for iPS Cell Research and Application (CiRA), Kyoto University, Kyoto, Japan; University of Kansas Medical Center, UNITED STATES

## Abstract

To realize human induced pluripotent stem cell (hiPSC)-based cardiac regenerative therapy, evidence of therapeutic advantages in human-sized diseased hearts are indispensable. In combination with an efficient and simultaneous differentiation of various cardiac lineages from hiPSCs and cell sheet technology, we aimed to generate clinical-sized large cardiac tissue sheets (L-CTSs) and to evaluate the therapeutic potential in porcine infarct heart. We simultaneously induced cardiomyocytes (CMs) and vascular cells [vascular endothelial cells (ECs) and vascular mural cells (MCs)] from hiPSCs. We generated L-CTSs using 10cm-sized temperature-responsive culture dishes. We induced myocardial infarction (MI) in micromini-pigs (15–25 kg) and transplanted the L-CTSs (Tx) 2 weeks after MI induction (4 sheets/recipient) under immunosuppression (Tx: n = 5, Sham: n = 5). Self-pulsating L-CTSs were approximately 3.5cm in diameter with 6.8×10^6^±0.8 of cells containing cTnT^+^-CMs (45.6±13.2%), VE-cadherin^+^-ECs (5.3±4.4%) and PDGFRβ^+^-MCs (14.4±20.7%), respectively (n = 5). In Tx group, echocardiogram indicated a significantly higher systolic function of the left ventricle (LV) compared to that in sham control (Sham vs Tx: fractional shortening: 24.2±8.6 vs 40.5±9.7%; p<0.05). Ejection fraction evaluated by left ventriculogram was significantly higher in Tx group (25.3±6.2% vs 39.8±4.2%; p<0.01). Speckle tracking echocardiogram showed a significant increase of circumference strain in infarct and border regions after transplantation. Fibrotic area was significantly lower in Tx group (23.8±4.5 vs 15.9±3.8%; P<0.001). Capillary density in the border region was significantly higher in Tx group (75.9±42.6/mm^2^ vs 137.4±44.8/mm^2^, p<0.001). These data indicate that the L-CTS transplantation attenuated LV remodeling. L-CTSs potentially restore cardiac dysfunction of human-sized infarct heart.

## Introduction

Cardiovascular diseases remain a major cause of death and increasing the burden of health-care worldwide, especially in the Western world [1]. This health problem has raised enthusiasms to find new therapeutic options including cardiac regenerative therapy using stem cells as a new paradigm for severe cardiac disorders resistant to conventional therapies [2, 3].

Pluripotent stem cells (PSCs) [embryonic stem cells (ESCs) / induced pluripotent stem cells (iPSCs)] -derived defined cardiovascular cell populations are considered to serve as a novel cell sources for cardiac regenerative therapy by virtue of theoretically infinite proliferative potential of PSCs [4, 5] and novel capacity to differentiate into various cardiovascular cell populations including cardiomyocytes (CMs), vascular endothelial cells (ECs) and vascular mural cells (MCs) [6–8]. We have previously reported a combined method to efficiently induce various cardiovascular cell populations [8] and a cell sheet technology based on temperature-responsive culture surface [9] which enabled us to collect cells as a sheet structure suitable for transplantation experiments onto animal models. The transplantation of heart tissue-mimetic cell sheets including defined cardiovascular cell populations (cardiac tissue sheets; CTSs) for sub-acute myocardial infarction (MI) rat models using mouse ESC- and human iPSC-derived cardiovascular cell populations have consistently demonstrated an excellent functional recovery of cardiac functional deterioration after MI [8, 10]. Although these proof-of-concept studies in small animals may represent the potential effectiveness of CTSs for the functional recovery from cardiac injury and may open the door for the realization of cardiac regenerative therapy using the CTS technology, verification of the therapeutic potential in clinical scaled injured hearts similar to the human heart would be required for clinical application of this strategy.

In the present study, we hypothesized and verified that large-sized human iPSC (hiPSC)-derived CTSs can be generated by expanding the technology as practiced in small animal studies and the large-sized CTSs possesses therapeutic potentials in large-animal injured hearts comparable to the results obtained with small CTSs in small animal MI models.

## Materials and methods

All experimental procedures were approved by the Kyoto University Animal Experimentation Committee (Med Kyo 16138) and performed in accordance with the guidelines for Animal Experiments of Kyoto University, which conforms to Japanese law and *the Guide for the Care and Use of Laboratory Animals* prepared by the Institute for Laboratory Animal Research, U.S.A. (revised 2011). All animals are treated with humane care with appropriate anesthesia and analgesia.

### Differentiation of human iPSCs into cardiovascular cell populations

We used a hiPSC line; 201B6[4] for generating cardiovascular cell populations. The methods for culturing and passaging human iPSCs have been previously reported in detail [8]. Briefly, iPSCs were detached with Versene (0.48 mM EDTA solution; Life Technologies, Carlsbad, CA, USA) and plated onto Matrigel (growth factor reduced, 1:60 dilution; Life Technologies)-coated plates at a density of approximately 100,000 cells/cm^2^ in mouse embryonic fibroblast conditioned medium [MEF-CM; Dulbecco’s modified Eagle’s medium (DMEM) (Nacalai Tesque, Kyoto, Japan) containing 10% fetal bovine serum (FBS), 2 mM L-glutamine, and 1% non-essential amino acids (NEAA) (Life Technologies)] with 4 ng/ml bFGF (Wako Pure Chemicals Industries, Osaka, Japan) for 2 days before induction. Cells were covered with Matrigel (1:60 dilution) 1 day before induction. MEF-CM was replaced with RPMI+B27 medium (RPMI1640, 2 mM L-glutamine, B27 supplement without insulin; Thermo Fisher Scientific, Waltham, MA, USA) supplemented with 100 ng/ml of Activin A (R&D, Minneapolis, MN, USA) for 1 day, followed by 10 ng/ml BMP4 (R&D) and 10 ng/ml bFGF for 3 days without culture medium change. At 5 days of differentiation, the culture medium was replaced with RPMI+B27 medium with 50 ng/ml VEGF_165_ (Wako Pure Chemicals Industries), and culture medium was refreshed every other day.

### Human iPSC-derived large cardiac tissue sheet (L-CTS) formation

Cardiovascular cells at 15 days after differentiation were dissociated by incubation with Accumax (Innovative Cell Technologies, San Diego, CA, USA) and plated onto a gelatin-coated 10-cm temperature-responsive culture dish (UpCell; CellSeed, Tokyo, Japan) at 10 x 10^6^ cells/dish with 17 ml of αMEM (Life Technologies) with 10% FBS, 50 ng/ml VEGF_165_ and Y-27632 (10 μM; Wako Pure Chemicals Industries), and culture medium was refreshed with 17 ml of RPMI+B27 medium with 50 ng/ml VEGF_165_ after 2 days. After another 2 days of culture (4 days in total; differentiation day 19), the cell sheets were collected. For detachment of cell sheets, the culture dish was moved to room temperature. Within 15–30 minutes, cell sheets detached spontaneously as L-CTSs.

### Flow cytometry

Flow cytometry for L-CTSs was carried out as previously described [8]. In brief, a part of L-CTSs was cut by sterile scissors and dissociated by incubation with Accumax, and were stained with one of the surface markers listed as follows: anti-PDGFRβ conjugated with phycoerythrin (PE), clone 28d4, 1:100 (BD, Franklin Lakes, USA); anti-VE-cadherin conjugated with fluorescein isothiocyanate (FITC), clone 55-7h1, 1:100 (BD). To eliminate dead cells, cells were stained with LIVE/DEAD fixable Aqua dead cell staining kit (Invitrogen, Eugene, OR, USA). For cell surface markers, staining was carried out in PBS with 5% FBS. For intracellular proteins, staining was carried out on cells fixed with 4% paraformaldehyde in PBS. Cells were stained with anti-cardiac isoform of Troponin T (cTnT) (clone 13211, Thermo Fisher Scientific) labeled with Alexa-488 using Zenon technology (Invitrogen) (1:50). The staining was performed in PBS with 5% FBS and 0.75% Saponin (Sigma, St. Louis, MO, USA). Stained cells were analyzed and sorted on an AriaII flow cytometer (BD). Data were collected from at least 5,000 events. Data were analyzed with DIVA software (BD).

### Calcium transient analysis

For calcium transient analyses, L-CTSs were attached on culture dish bottom, cultured with RPMI1640 medium containing 2mM L-glutamine, 10% FBS and VEGF_165_ for 2 days and loaded with 5 μM Quest Fluo-8 (ABD Bioquest, Inc. Sunnyvale, USA) for 1 hour. Fluo-8 fluorescence (excitation at 495±610 nm and emission at 535±620 nm) of beating sheet was measured every 64 msec and visualized using Biorevo BZ-9000 (Keyence, Osaka, Japan). The measurements were carried out at room temperature.

### Porcine models and protocols for MI induction and L-CTS transplantation

Ten female micro mini-pigs weighing 15 to 25 kg (Fuji Micra Inc, Shizuoka, Japan) were used. The pigs were randomly divided into 2 groups; sham control (n = 5) and L-CTS transplantation (Tx) (n = 5 for each). For all surgical procedures, the pigs were treated with an intramuscular injection of ketamine hydrochloride (20 mg/kg), xylazine hydrochloride (2 mg/kg) and atropine sulfate (0.5 mg) prior to the induction of general anesthesia, then general anesthesia was induced with Propofol (20 mg) intravenously through an ear vein and maintained with 5L/min of O_2_ and sevoflurane (1–2%). The pigs were intubated with a cuffed endotracheal tube (7.0 mm). Cefazolin sodium (500 mg) was injected intravenously administered before skin incision. Xylazine hydrochloride (2 mg/kg) was additionally administrated in every 30 minutes for analgesia. The animals were positioned in the right lateral decubitus position and an electrocardiogram was monitored. A 5F size short sheath was placed in the common femoral artery and blood pressure was monitored through the sheath.

For MI induction, the pericardial space was exposed through left thoracotomy at the 4^th^ intercostal space. An ameroid constrictor (Neuroscience Inc., Tokyo, Japan) was placed around the left anterior descending coronary artery (LAD) just distal of the branching of the first diagonal branch, and the most proximal portion of the first diagonal branch was permanently ligated. The pericardium, muscle and skin were closed by layers.

Two weeks after MI induction, the pigs were subjected to L-CTS transplantation or sham operation. In Tx group, 4 L-CTSs were put on the MI area and manually unfolded to make whole MI area covered by the L-CTSs. The L-CTSs could be stably placed onto the epicardium without sutures. The chest and pericardium were closed 15 minutes after L-CTS transplantation. In the sham group, the chest was closed 15 minutes after thoracotomy without L-CTS transplantation.

After 4 weeks of observation and functional evaluation, the pigs are euthanized by intravenous bolus injection of potassium chloride (1–2 mmol/kg) and the hearts are harvested.

### Transthoracic echocardiogram

Cardiac function was assessed using an iE33 system (Philips Medical Systems, Andover, MA) provided with a 4.0-MHz transducer (Philips Medical Systems, Andover, MA). Echocardiographic measurements were performed as previously described[8].

For speckle tracking strain echocardiography, we obtained echocardiographic images in 2 levels of short axis (base / mid) of the left ventricle (LV) and analyzed the data using QLAB Ultrasound Cardiac Analysis software (Philips) with the electrocardiogram-gated mode. The images were tagged as 17-segments (American Heart Association) [11] and the circumference strain of each segment was automatically measured.

### Left ventriculogram

The animals are positioned as right lateral decubitus position under general anesthesia. A pig-tail catheter was introduced to LV through a 5F sheath at the femoral artery. Left ventriculogram was performed with a fluoroscopic apparatus (OEC 9800 Plus; General Electric, Fairfield, CT) with a manual injection of Iopamidol.

Three serial images of end-systolic and end-diastolic phase were obtained, and the area of LV area (visualized by contract agent) was visualized by Image J software[12]. The average of data from three serial images are calculated as systolic or diastolic area and ejection fraction was calculated by the area. In sham control at PreMI, one data was missing because of a technical failure.

### Histological analysis

Heart tissues were collected just after euthanasia and divided into 4 parts. The samples were fixed in 10% formalin and subsequently embedded in paraffin. Five sections with 6 μm thickness were made at 50 μm intervals along the short axis from the center of the MI area for each animal and examined for Masson trichrome staining, Periodic acid-Schiff (PAS) staining and immunofluorescence staining. For immunofluorescent staining, sections were treated with Protein Block Serum Free (DAKO, Carpinteria, CA) and incubated for 60 min with primary antibodies at room temperature. For capillary density (capillary number/mm^2^), 20 random views for every rat hearts evenly including border region and central region of MI (×400, original magnification) were selected from vWF-stained sections, and the number of capillaries was manually counted in each view. For vWF staining, a rabbit polyclonal antibody (DAKO) was used as the primary antibody (1:800) followed by LSAB2 Kit/horseradish peroxidase (HRP) (diaminobenzidine; DAB) (DAKO) according to the manufacturer’s instructions. For the assessment of engrafted human cells, anti-Ku80 antibody (Abcam, Cambridge, UK) was used as the primary antibody. All immunostained sections were photographed and calculated with Biorevo BZ-9000 (Keyence).

### Statistical analysis

At least three independent experiments were performed. All data analyses were performed using JMP version 11.2.0 (SAS Institute, Cary, NC, USA). Statistical analysis of the data was performed with ANOVA for >2 groups (Tukey’s test as post-hoc), and unpaired t-tests for 2 groups. p<0.05 was considered significant. Values are reported as means ± SD.

## Results

### Generation of L-CTS

**Fig 1:**
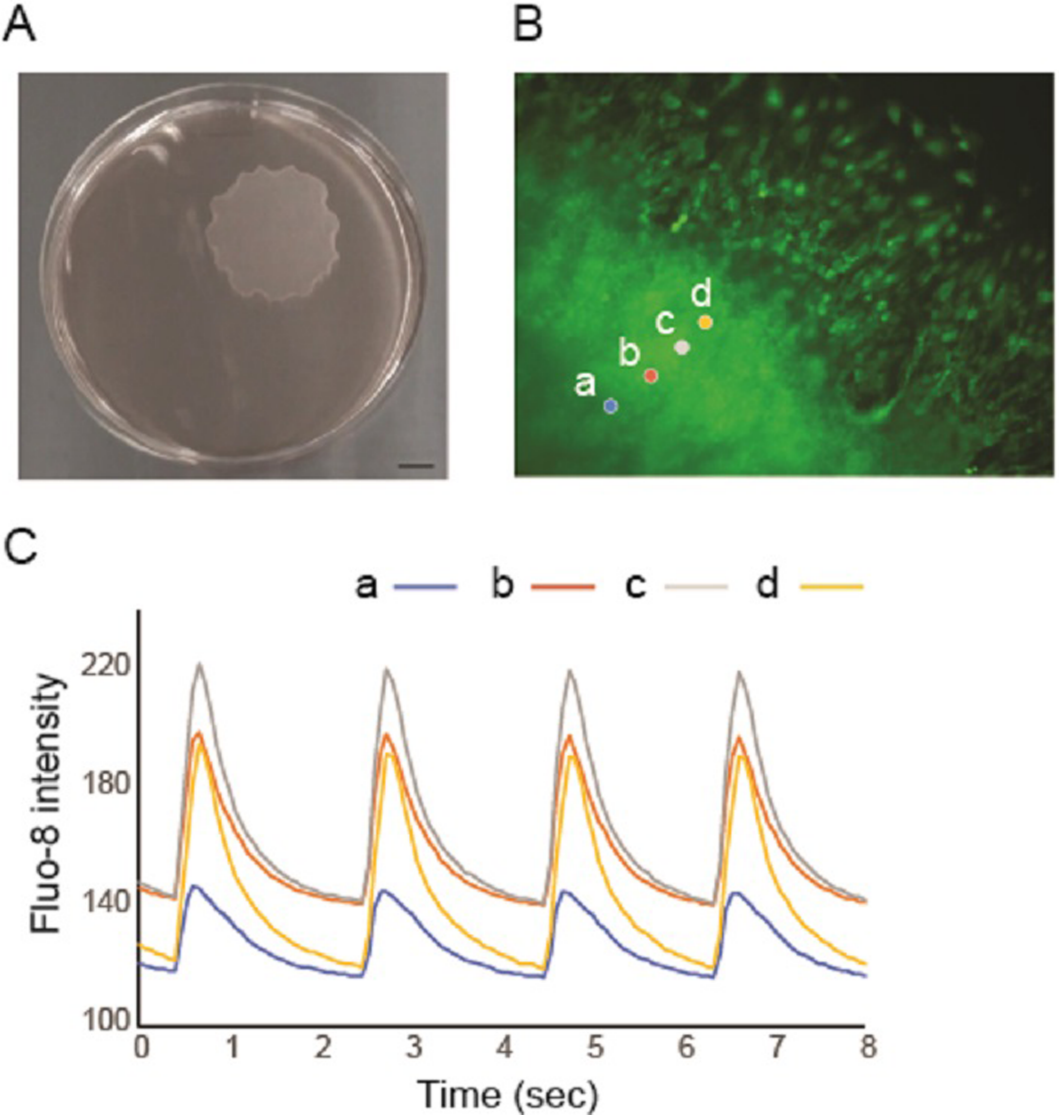
Generation of large cardiac tissue sheets (L-CTSs). (A) Optimal view of the L-CTS placed in a 10cm-sized UpCell dish. Scale bar = 1 mm. (B) (C) Calcium transient of L-CTSs. (B) Representative Fluo-8 image of L-CTS (see also S1 video) and region of interests (ROIs). Magnification ×100. (C) Chronologic intensity change of Fluo-8. Note that the peak timings of 4 different ROIs are almost same chronologically.

First, we attempted to generate clinical-scaled L-CTSs. We simultaneously induced cardiovascular cell populations from hiPSCs using a monolayer high-density serum-free culture method which we have previously reported [8]. We re-cultured the induced cardiovascular cell population onto 10-cm size temperature-responsive culture dishes. After 4 days of culture, we successfully collected self-pulsating L-CTSs. The size of the L-CTS was approximately 3.5 cm in diameter (Fig 1A). The L-CTSs consisted with 6.8×10^6^±0.8 (n = 5) of cells containing cTnT^+^-CMs (45.6±13.2%), VE-cadherin^+^-ECs (5.3±4.4%) and PDGFRβ^+^-MCs (14.4±20.7%) (n = 5). Regular calcium transients were observed throughout the L-CTSs along with spontaneous beating (Fig 1B and 1C and S1 video) indicating that cardiac tissue-like structure including CMs and vascular components was successfully generated from hiPSCs.

### Functional recovery after L-CTS transplantation onto a porcine MI model

We induced MI in porcine hearts. The implantation of ameroid constrictor at the proximal region of the left anterior descending artery and a permanent ligation of the first diagonal branch apparently reduced wall motion of anterior to antero-lateral region at 2 weeks after MI induction. We evaluated cardiac function of the pigs by echocardiogram before and after MI induction and confirmed that the procedures successfully reduced systolic function of the pigs [Before MI vs 2 weeks after MI: fractional shortening (FS); 46.0±8.1 vs 26.6±5.5, P<0.001 / fractional area change (FAC); 65.2±10.6 vs 47.1±10.4, P<0.001, n = 10].

**Fig 2:**
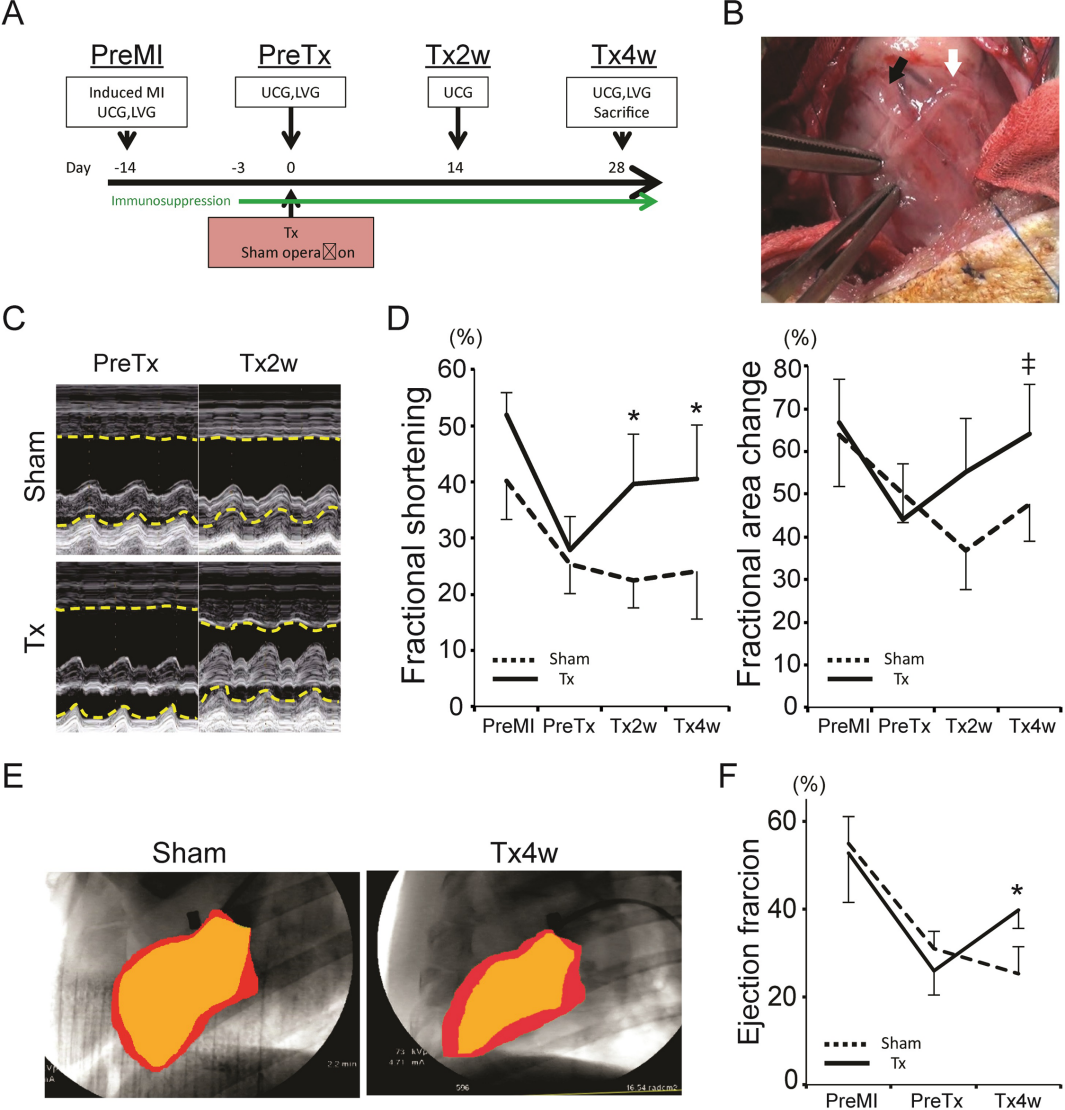
Transplantation of L-CTSs onto porcine infarct hearts. (A) Time course of the animal experiments. MI, myocardial infarction; UCG, ultrasound cardiogram (echocardiogram); LVG, left ventriculogram; Tx, treatment. (B) Surgical view during L-CTS transplantation. The first L-CTS is shown at black arrow and the second L-CTS is shown at white arrow. (C) M-mode view of the echocardiogram. Dotted yellow lines indicate the endocardial boundary of anterior or posterior wall. PreTx, before transplantation; Tx2w, 2 weeks after transplantation. (D) systolic function after transplantation. *p<0.05 vs Sham, ‡p<0.01 vs PreTx. (E) Left ventriculogram at 4 weeks after transplantation. Red: end-diastolic phase, orange: end-systolic phase. (F) Ejection fraction calculated by left ventriculogram. *p<0.05 vs Sham. PreMI, before MI induction.

We divided the 14 pigs into 2 groups [sham operation / transplantation (Tx)] (n = 7 for each). For Tx group, we carefully dissected the adhesion of the heart surface and transplanted the L-CTSs onto the porcine MI model which covered almost whole region of the infarcted area (Fig 2A and 2B) (4 CTSs / pig). We observed the pigs for 4 weeks after the L-CTSs transplantation. Four pigs died immediately after induction of general anesthesia for functional evaluation after 2 or 4 weeks after surgery due to acute deterioration of heart failure (sham: 1 at 2 week, 1 at 4 week / Tx: 1 at 2 week, 1 at 4 week). Survived 10 pigs are included in the study group (n = 5 for each). There was neither animal sudden death possibly due to lethal arrhythmia, nor tumor formation during the observation period. Echocardiographic examination revealed that the transplantation of L-CTSs restored wall motion of transplanted region (Fig 2C) and showed significantly higher systolic functional parameters compared to those in sham group at 4 weeks after surgery, or those before transplantation (sham vs Tx: FS; 24.2±8.6 vs 40.5±9.7%; P = 0.021) (PreTx vs Tx4w: FAC; 44.1±13.2 vs 64.1±11.6%; P = 0.0093) (Fig 2D). To evaluate detailed myocardial functional recovery, we performed regional wall motion analysis. The ratio of contraction change evaluated by circumference strain was significantly improved in some of the transplanted region (Tables 1 and 2). We also performed left ventriculogram and found that the ejection fraction in transplanted group at 4 weeks after transplantation showed higher tendency compared to that before transplantation (PreTx vs Tx4w: 25.9±5.5 vs 39.8±4.2%; p = 0.056), and was significantly higher compared to that in sham group at 4 weeks after surgery (Sham vs Tx: 25.3±6.2 vs 39.8±4.2% %; p = 0.031) (Fig 2E and 2F). We did not observe any abnormality in rhythm during the evaluations by echocardiogram and left ventriculogram.

**Table 1:** Regional wall motion evaluated by circumference strain at base region.

		Base					
		Infero-Septal	Antero-Septal	Anterior*	Antero-Lateral	Infero-Lateral	Inferior*
Sham	PreMI (%)	21.5±15.0	21.5±14.6	20.5±10.8	18.3±2.6	24.8±9.3	20.8±10.1
	PreTx (%)	23.0±6.0	18.4±11.7	16.2±8.0	15.6±5.4	17.8±8.2	14.8±10.0
	Tx2w (%)	24.8±2.7	15.6±9.4	17.2±8.5	19.0±8.5	19.2±8.3	21.8±5.6
	Tx4w (%)	20.4±7.0	22.4±5.8	15.0±6.5	20.6±3.4	20.0±5.6	18.4±5.9
	Tx4w/PreTx(ave.)	0.89	1.22	0.93	1,32	1.12	1.24
Tx	PreMI (%)	34.3±4.9	23.5±7.9	28.3±11.4	27.8±3.0	31.5±3.3	25.5±8.3
	PreTx (%)	23.0±6.2	18.8±8.7	13.6±5.9	19.8±4.1	21.8±9.2	25.2±4.4
	Tx2w (%)	23.4±5.6	26.2±4.0	21.8±4.3	19.6±7.9	21.8±7.6	22.4±6.9
	Tx4w (%)	33.0±11.0	27.6±8.1	24.8±3.3	27.0±6.6	30.4±8.4	25.4±6.2
	Tx4w/PreTx(ave.)	1.43	1.47	1.82	1.36	1.39	1.01

**Table 2:** Regional wall motion evaluated by circumference strain at mid region.

		Mid					
		Infero-Septal	Antero-Septal	Anterior†	Antero-Lateral*	Infero-Lateral	Inferior
Sham	PreMI (%)	35.5±11.9	23.8±16.1	25.0±15.3	28.5±10.9	32.0±15.8	28.8±17.1
	PreTx (%)	26.4±8.3	19.2±4.7	11.6±3.8	14.4±3.5	21.4±4.4	18.0±6.7
	Tx2w (%)	22.8±10.0	11.6±5.2	10.8±4.1	13.0±6.5	16.6±10.6	15.6±9.7
	Tx4w (%)	23.6±7.7	17.6±4.4	15.2±6.5	18.8±4.2	23.2±9.0	22.2±6.2
	Tx4w/PreTx(ave.)	0.89	0.92	1.31	1.31	1.08	1.23
Tx	PreMI (%)	34.5±9.5	29.0±8.0	37.0±7.1	32.3±7.1	29.8±15.9	32.3±7.6
	PreTx (%)	23.8±4.4	14.0±8.3	11.6±9.4	17.0±6.6	20.2±3.1	25.8±8.0
	Tx2w (%)	34.6±8.6	19.2±8.3	20.6±3.5	24.8±7.6	23.2±4.7	23.0±5.4
	Tx4w (%)	38.0±1.9	21.2±6.6	21.2±8.1	24.0±4.1	30.0±6.1	26.2±4.4
	Tx4w/PreTx(ave.)	1.60	1.51	1.83	1.41	1.49	1.02

*p<0.05 (2-way ANOVA; PreTX, Tx2w and Tx4w),

†p<0.05 (Tukey’s test; PreTx vs Tx4w).

Absolute values are shown.

### Attenuated left ventricular remodeling after L-CTS transplantation

**Fig 3:**
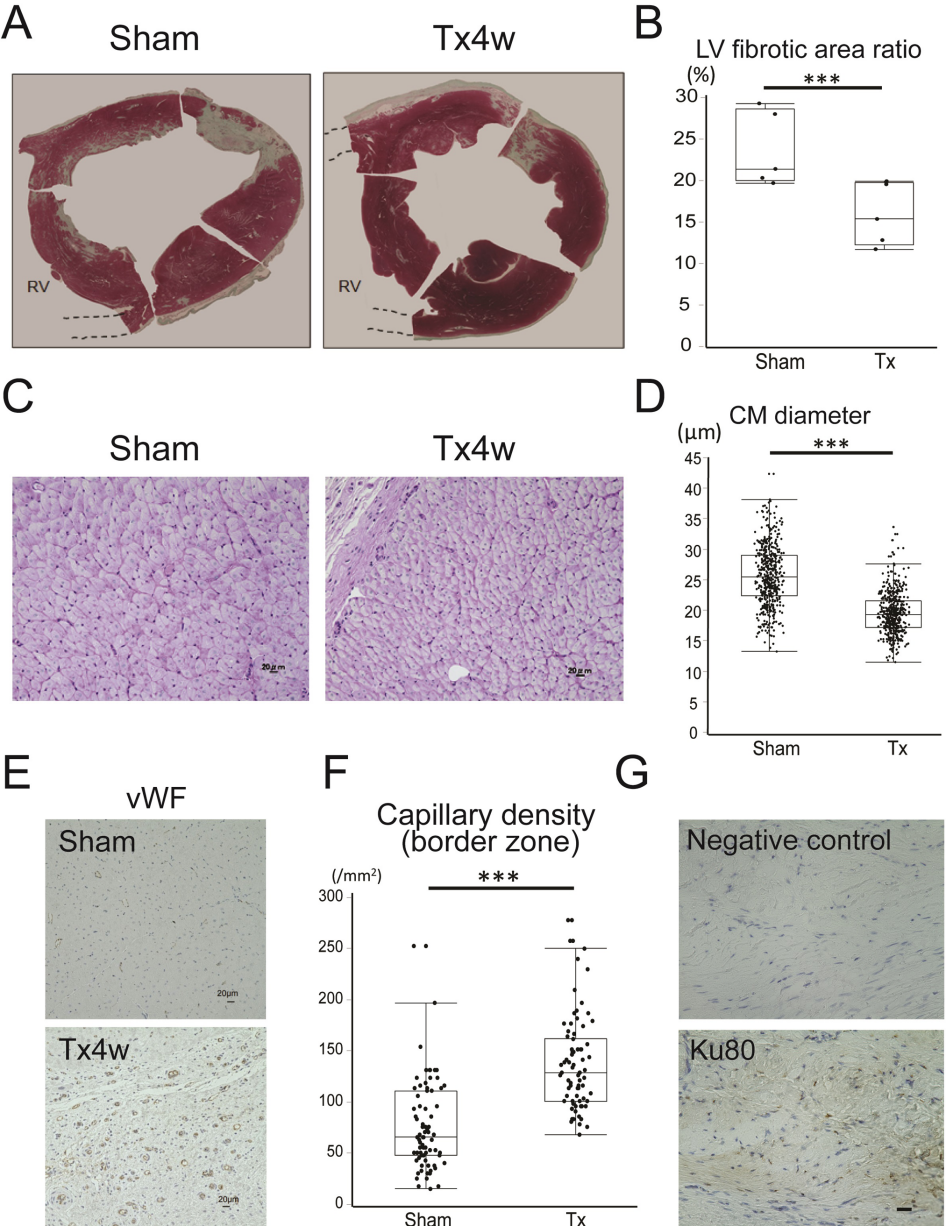
Attenuation of LV remodeling after L-CTS transplantation. (A) Representative Masson’s trichrome staining at 4 weeks after transplantation. RV, right ventricle (removed). (B) The ratio of fibrotic area to the whole heart area. ***p<0.001. (C) Representative PAS staining of border region at 4 weeks after transplantation. Scale bar = 20 μm. (D) Cardiomyocyte diameter. ***p<0.001. (E) Representative vWF staining 4 weeks after L-CTS transplantation (brown). Scale bar = 20 μm. (F) Capillary density at border region. ***p<0.001. (G) Representative Ku80 staining (brown). Scale bar = 20 μm.

We evaluated the extent of fibrosis in the hearts of both groups. Fibrotic area evaluated by Masson’s trichrome stain revealed that the fibrotic area was significantly lower in the transplanted group compared to that in the sham group (Sham vs Tx: 23.8±4.5 vs 15.9±3.8%; p<0.001) (Fig 3A and 3B). Next, we evaluated the size of cardiomyocytes in the border region of MI using PAS stain. The diameter of cardiomyocytes was significantly smaller in transplantation group compared to that in the sham group (Sham vs Tx: 25.6±4.9 vs 19.5±3.4 μm; p<0.001) (Fig 3C and 3D). We also evaluated capillary density in the border region and found that the capillary density was significantly higher in the treatment group than in sham group (75.9±42.6 vs 137.4±44.8 /mm^2^; p<0.001) (Fig 3E and 3F). These data indicate that the L-CTS transplantation induced neovascularization in the border region of MI which led to attenuation of cardiomyocyte hypertrophy and global LV remodeling. We evaluated the survival of transplanted cells using Ku80 immunostaining which is a specific marker of human cells [13] at 4 weeks after transplantation. Although we could find a small fraction of Ku80-positive engrafted cells, the amount was relatively small (Fig 3G).

## Discussion

In the present study, we have successfully generated L-CTSs suitable for human-sized hearts with a simple modification of previously reported method using hiPSC-derived various cardiac cells and temperature-responsive culture surface [8]. Further, we have verified the therapeutic effects of the L-CTSs on infarcted porcine hearts mainly through paracrine mechanisms such as angiogenetic, anti-fibrosis and/or the attenuation of cardiomyocyte hypertrophy, all of which could have attributed to the attenuation of LV remodeling.

Although numerous reports have proven the potential of cardiac regenerative therapy using hiPSCs including the therapeutic effectiveness and safety [14], the proof-of-concept of the therapeutic potential for human heart could only be obtained from experiments using human-sized hearts. In the present study, we employed a porcine sub-acute MI model, and have successfully demonstrated the therapeutic potential of L-CTSs for diseased heart which may be advantageous for clinical application of this cell-based therapeutic material in the future.

The therapeutic mechanism in the present porcine study was mainly mediated by paracrine mechanisms considering small engrafted cell numbers which cannot be attributed to the reinforcement of mechanical ventricular contraction. It is well recognized that the attenuation of cardiomyocyte hypertrophy and cell death at MI border region is essential for the protection of infarction expansion [15] leading to LV remodeling in the chronic phase of MI [16, 17]. Considering increased capillary density in the border region which might have prevented the hypertrophic cardiomyocytes with an increase of oxygen demand from cell death, angiogenic stimuli from the L-CTSs in the present study would have contributed to suppressing the progression of LV remodeling as mentioned above [10, 15]. Further investigations to verify the capacity of L-CTSs on vascular network formation *in vitro* would strongly support the ability of L-CTSs on neovascularization observed in the present *in vivo* study which would be conducted in our future studies aiming for the elucidation of therapeutic mechanisms of L-CTSs.

Although the therapeutic mechanism is similar to that observed in rat MI experiments which we have previously published [8, 10], the therapeutic effect and engraftment efficiency was rather smaller than that shown in previous rat studies. This is most likely due to the relatively smaller number of engrafted cells compared to those in rat experiments. Although the body weight of pigs in the present study was >50 fold higher compared to rat models in the previous study[8], delivered cell number was just around 12-folds larger (2.2×10^6^ cells vs 2.7×10^7^ cells). The optimal dose of cells which shows therapeutic effects for various pathological condition of the host heart should be further investigated. In this regard, utilizing bioengineered 3-dimensional thickening of the cell sheets using gelatin hydrogel microspheres which have been reported to support oxygen and nutrient supply for the whole structure of considerably larger thickness would be advantageous [18] considering that the transplantation experiments in the current study were performed as a single-layered sheet application.

We did not observe any animal death during the experimental period of L-CTS transplantation except the timings of the induction of general anesthesia. This result indicates that the animals were free from lethal arrhythmia. It is crucial to further investigate the possibility of the occurrence of arrhythmia using telemetry or Holter electrocardiogram to validate the safety of this therapeutic method before clinical application. Another requirement before clinical application is to check the tumorigenicity after the L-CTS transplantation. The usage of genomic integration-free iPSCs [19] would probably reduce the risk of tumorigenicity due to the lower possibility of genomic integration and further experiments using genomic integration-free iPSCs would be required as a pre-clinical safety test.

We have only checked the therapeutic potential in a specific phase of MI (sub-acute MI). Although it might be challenging to use another pathological condition such as ischemic cardiomyopathy or dilated cardiomyopathy using porcine models, the creation of disease models and validation of the therapeutic potential of L-CTSs in such models should be attempted in the future.

In the present study, we have employed ultrasound-based cardiac functional evaluations. Although our data include both global function and regional wall motion in every segment of LV wall which provided us comprehensive functional data, we may need to use other modalities including cardiac computed tomography or magnetic resonance imaging [20] to precisely verify cardiac function. For functional evaluation and safety testing, we should also observe for a longer period. We also recognize that the therapeutic potential would not be sufficient to prevent acute onset of heart failure considering that same number of animals in both group died just after the induction of general anesthesia (2 out of 7 for each). Improvement of the therapeutic strategy using L-CTSs would be further required for a broad clinical application of the present method. Next, we performed histological analyses only at one-time point (4 weeks after transplantation). Evaluations at several time intervals after the L-CTS transplantation may be helpful to further elucidate the mechanism of functional restoration.

## Conclusion

Here we have shown a proof-of-concept for the realization of hiPSC-based cell therapy using cell sheet technology in human-sized hearts. Verification of therapeutic safety will be necessary to further develop this strategy for the clinical application.

## Supporting information

S1 Video Calcium oscillation of the L-CTS. The chronological change of the intracellular calcium concentration according to spontaneous beating is visualized as the change of fluorescence intensity.
(MP4)
